# Surgical strategy to prevent cardiac injury during reoperation in infants

**DOI:** 10.1186/1749-8090-3-10

**Published:** 2008-02-28

**Authors:** Christopher J Knott-Craig, Steven P Goldberg, James K Kirklin

**Affiliations:** 1 Division of Cardiothoracic Surgery, University of Alabama at Birmingham, Birmingham, Alabama, USA

## Abstract

**Introduction:**

Simplified Aortic Cannulation (SAC), wherein the innominate artery is used as the arterial inflow site rather than the ascending aorta, has proved to be a useful technique for arterial cannulation especially for small neonates undergoing complex cardiac operations. Since few technical options are available for re-entry cardiac injuries in small infants, we postulate that this technique may be equally helpful in those situations.

**Case Presentation:**

We employed SAC in 4 infants undergoing reoperative cardiac surgery (prior Norwood, n = 2; prior arterial switch operation with suprasystemic pulmonary artery pressures after a Le Compte maneuver, n = 1; prior Ebstein's anomaly, n = 1). In all cases the innominate artery was exposed at the level of the supra-sternal notch, and a 3.5 mm expanded polytetrafluoroethylene (ePTFE) graft was anastomosed to the innominate artery (n = 3), and a 10 French cannula inserted into the graft for whole-body perfusion. Right atrial cannulation was obtained by dividing the anterior aspect of the diaphragm at the level of the xiphisternum, gaining easy access to the right atrial-inferior vena cava junction, without separating the sternal edges.

**Discussion and Evaluation:**

All four infants successfully underwent their operations using SAC. In one case (2^nd ^stage palliation for hypoplastic left heart syndrome) a cardiac injury occurred upon sternal reentry, but utilizing SAC, this was repaired without consequence.

**Conclusion:**

Simplified aortic cannulation and direct right atrial cannulation may be obtained without dividing the sternum in complex reoperative infant surgeries, without making additional incisions. This may be life-saving in reoperative cardiac injuries in small infants.

## Introduction

Simplified Aortic Cannulation (SAC), wherein the innominate artery is used as the arterial inflow site rather than the ascending aorta, has made a significant impact on the conduct of complex operations for congenital heart disease, such as the Sano-Norwood operation, and cardiac operations in very small neonates or those with diminutive ascending aortas. [1,2]

In young infants requiring resternotomy, the femoral vessels are too small to initiate cardiopulmonary bypass, and the neck has usually been accessed for venous lines. Consequently, few alternatives exist for the congenital cardiac surgeon, in cases at high risk for re-entry cardiac injury, or, in the event of uncontrollable re-entry injury having occurred. Some advocate more proximal, but still "peripheral" cannulation, such as iliac access via retroperitoneal exposure, but this is requires a separate incision, and is time-consuming [3].

In the current era, there is an increasing volume of young infants aged 2–4 months who require resternotomy, often with systemic pressures in their right ventricle, (e.g., hypoplastic left heart syndrome [HLHS], coming for 2^nd ^stage), or with right ventricular-to-pulmonary artery conduits in close proximity to the back of the sternum (s/p Sano-Norwood operation), or in whom the ascending aorta is difficult to access expeditiously (s/p arterial switch operation [ASO] with LeCompte maneuver).

We describe a very useful technique which may be life-saving in these situations. We propose to broaden the indications for SAC to include reoperations, as the cannulation can be performed prior to even initiating the resternotomy, minimizing the effects of unintended entry into mediastinal structures.

## Case Presentation

**Figure 1:**
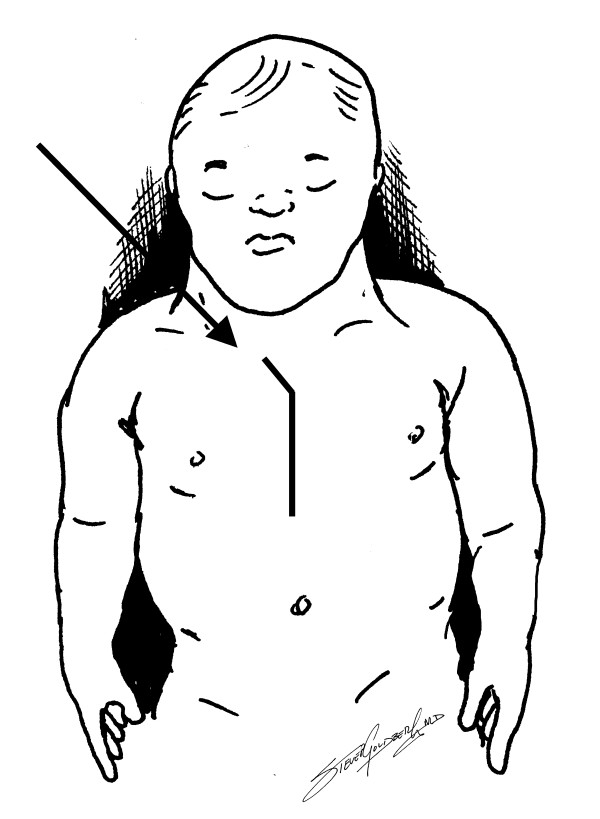
Rightward extension of skin incision in preparation for Simplified Aortic Cannulation (arrow).

**Figure 2:**
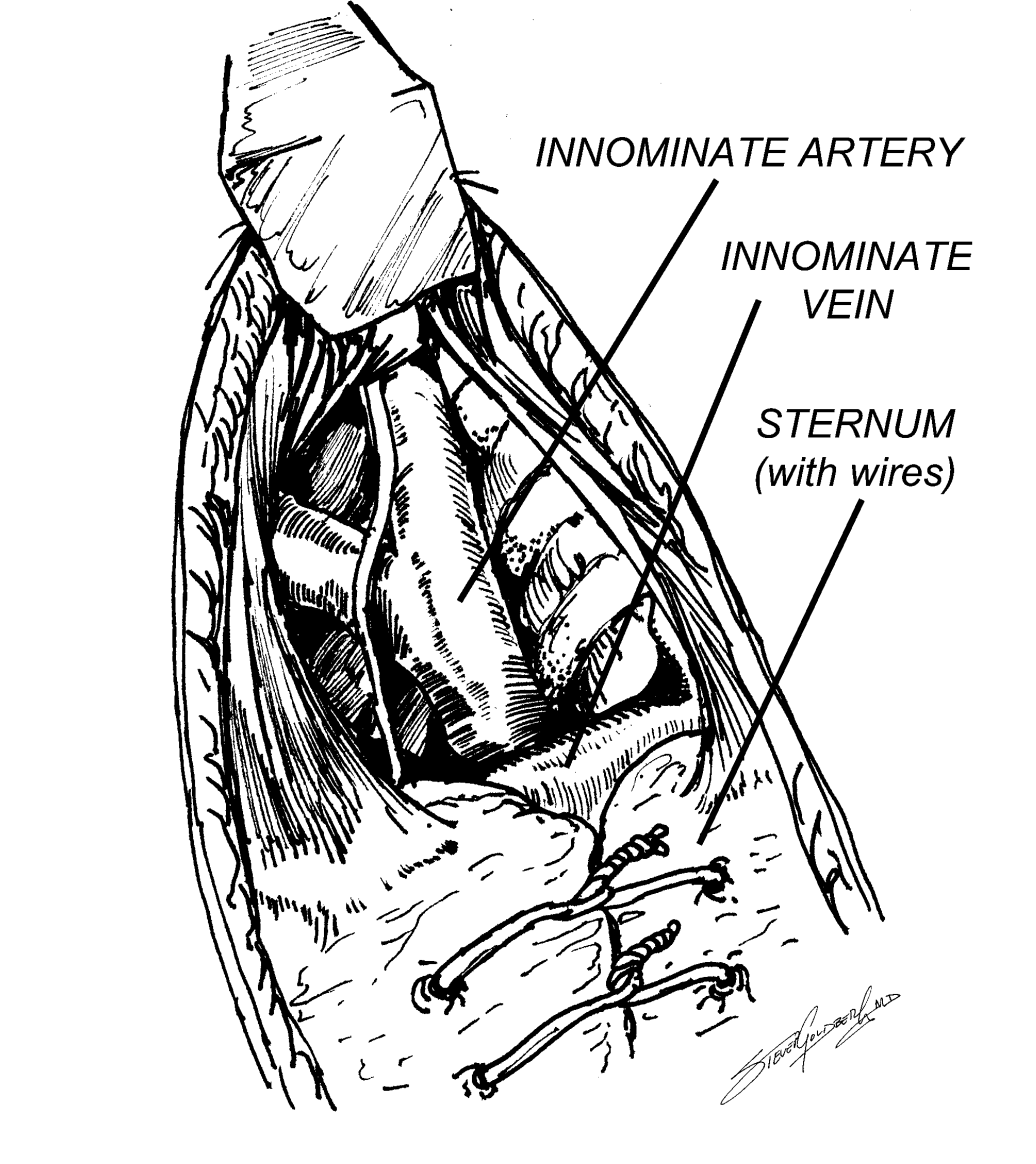
Dissection of innominate artery cephalad to innominate vein.

**Figure 3:**
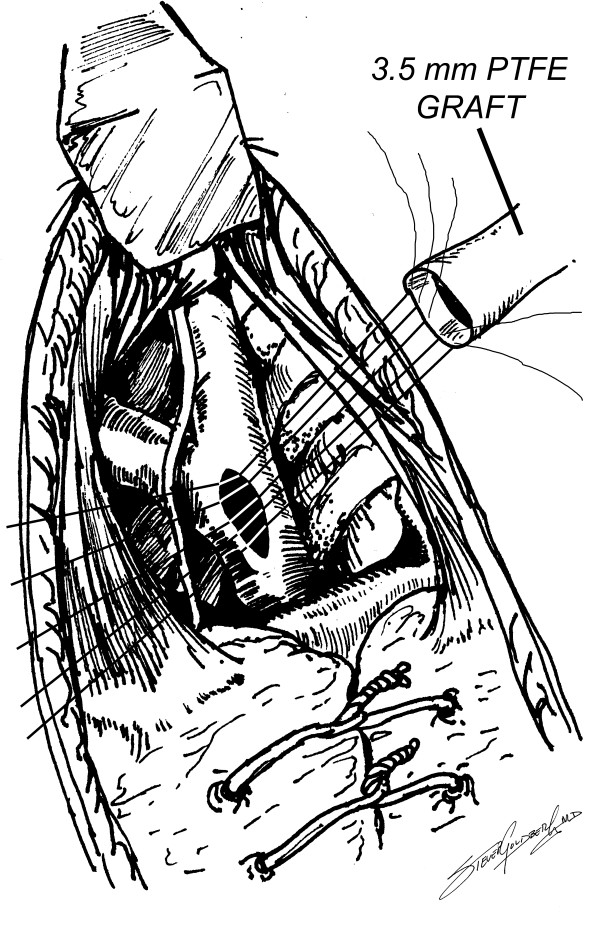
Anastomosis of PTFE graft to innominate artery (PTFE = polytetrafluoroethylene).

**Figure 4:**
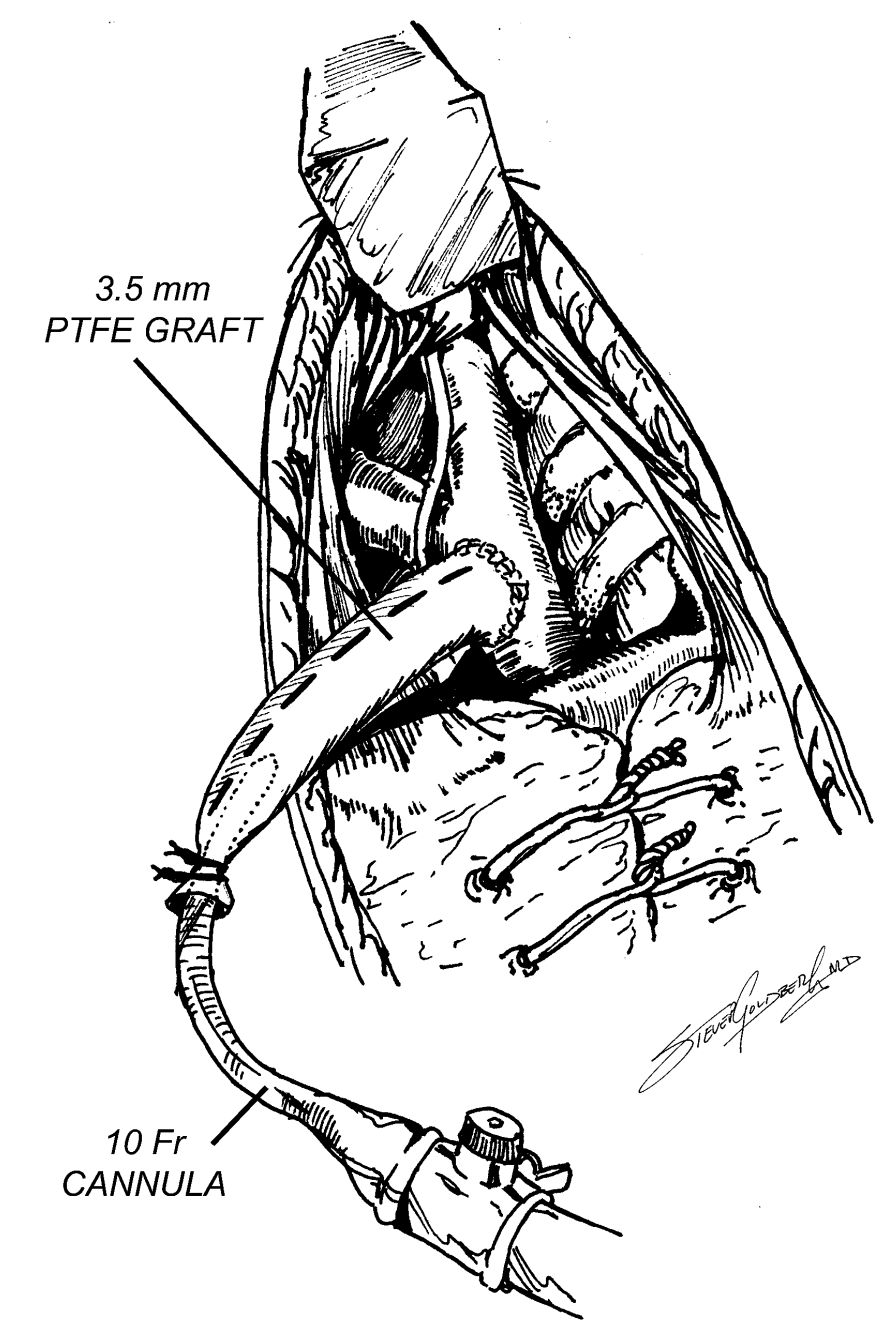
Cannulation of the graft for arterial inflow.

The standard technique of SAC has previously been described by us in detail in the literature [1]. Specifically, in the setting of a reoperation, the initial skin incision over the previous scar is extended to the supra-sternal notch and slightly to the right. (Figure 1). This will allow easy exposure of the innominate artery in the superior mediastinum cephalad to its crossing behind the innominate vein (Figure 2). The artery is dissected out above the innominate vein, and encircled by a vessel loop. A side-biting Castañeda clamp is applied, and a longitudinal arteriotomy is made. The patient can be systemically heparinized at this point. A 3.5 mm thin-walled expanded polytetrafluoroethylene (ePTFE) graft is anastomosed in an end-to-side fashion using a running 7-0 polypropylene suture (Figure 3). The graft is left long, and a 10-French arterial extracorporeal membrane oxygenation (ECMO) cannula is inserted into the free end of the ePTFE graft and secured with a silk tie; this is used for the arterial side of the cardiopulmonary bypass circuit, allowing full-flow whole-body perfusion (Figure 4). Alternatively, the innominate artery can be cannulated directly in the standard fashion.

**Figure 5:**
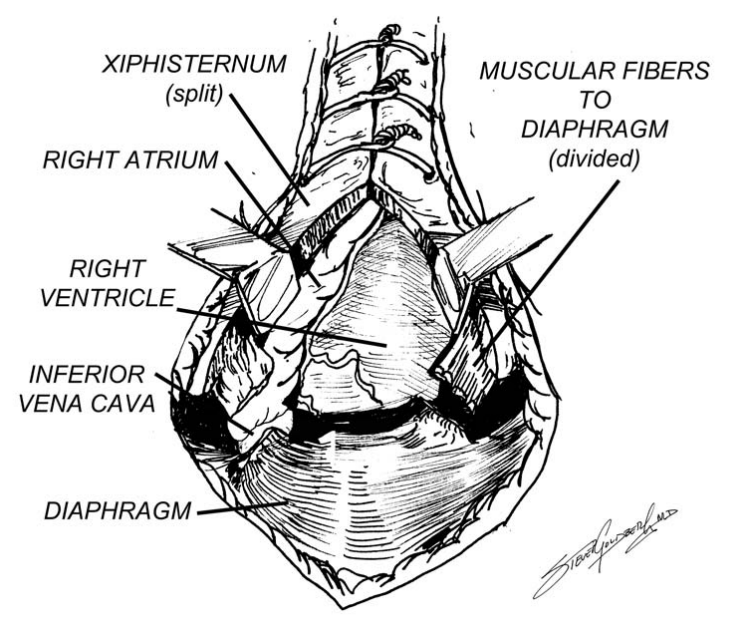
Exposure of the right atrium by dividing the xiphisternum and muscular fibers to the diaphragm.

**Figure 6:**
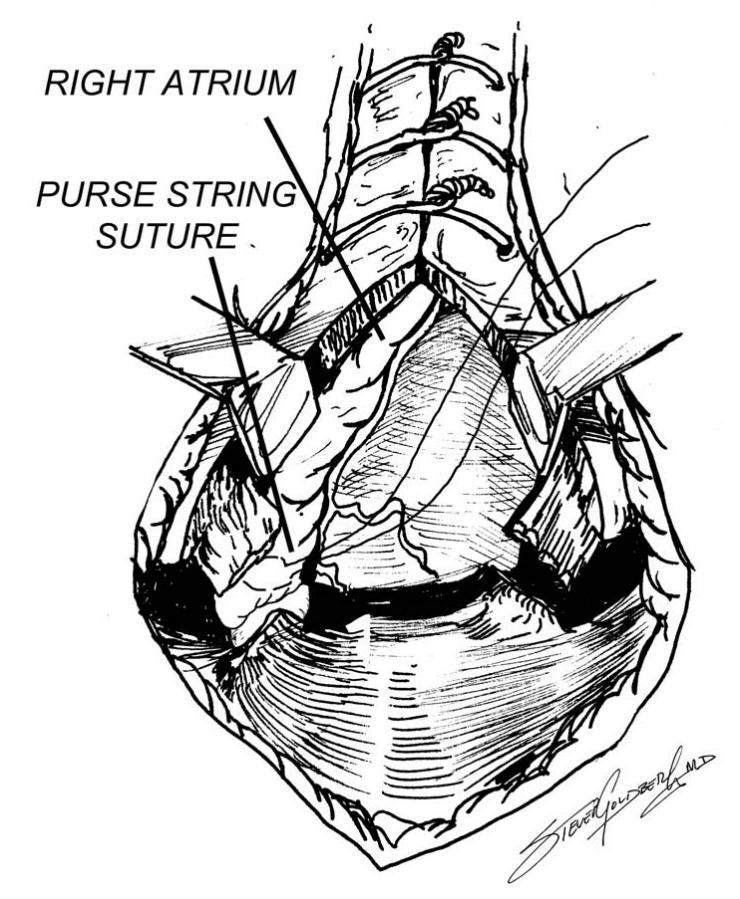
Placement of purse string suture in right atrium.

**Figure 7:**
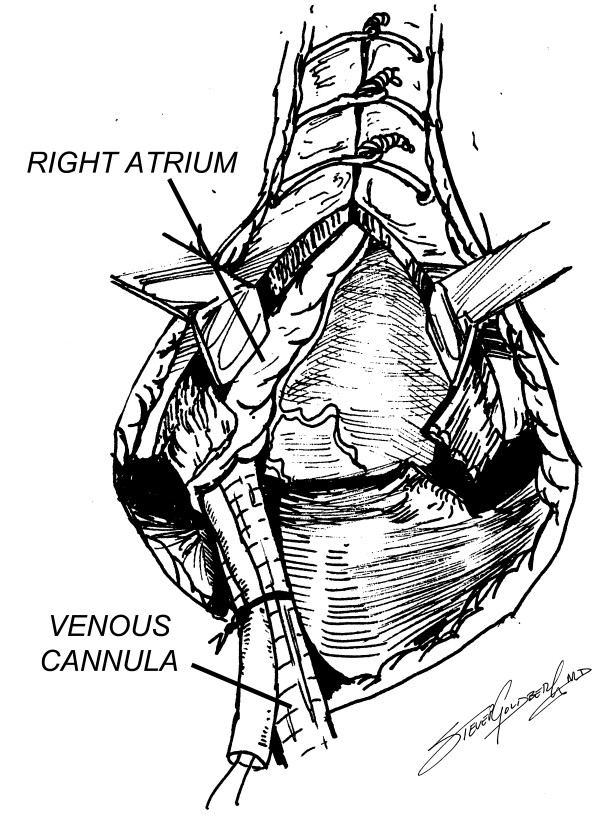
Venous cannulation for bypass.

Venous access for cardiopulmonary bypass can be easily achieved by extending the sternotomy incision to just below the xiphisternum, which is split. The anterior muscular fibers bringing the central portion of the diaphragm to the xiphisternum are divided with a Bovie, and the diaphragmatic surface of the right atrium can be very easily accessed through the upper centimeter or so of the diaphragm, since most often there are very few adhesions in this area (Figures 5, 6). A single venous cannula can be inserted (Figure 7), allowing the initiation of cardiopulmonary bypass (CPB), thereby decompressing the right heart, allowing the sternal reentry to be completed more easily. At the conclusion of the operation, a metal Weck clip is placed across the base of the PTFE graft parallel to the innominate artery, and the heparin is reversed.

## Discussion and Evaluation

We have successfully used this technique in 4 infants between January and May, 2007. Two patients (3 months old and 4 months old) had HLHS and were undergoing 2^nd ^stage palliation. In the first, the neoaorta was injured during reentry and CPB was initiated using this technique and the cardiac injury repaired. In the other, the Sano (right ventricle-to-pulmonary artery) conduit was embedded in the underside of the sternum, and could not be safely freed during the re-sternotomy. The third patient (1 year old) had undergone numerous previous operations elsewhere for complications of arterial switch operation with LeCompte maneuver for Taussig-Bing anomaly. The patient had been catheterized numerous times and the femoral vessels were at best tenuous. The patient had supra-systemic pressures in the pulmonary arteries which were in close proximity to the back of the sternum. This technique was utilized for this, the patient's fifth sternotomy. The last patient had a resternotomy for Ebstein's anomaly, and the huge, very thin-walled right atrium was not only adherent to the sternum, but completely obscuring the ascending aorta. This technique was successfully utilized in all four patients.

## Conclusion

We use Simplified Aortic Cannulation routinely for complex congenital heart surgery, especially in very small neonates and those with small ascending aortas, or in whom the ascending aorta needs repair (e.g., aorto-pulmnoary window). It allows good arterial access in situations where the aorta is either too diminutive to accept a sufficiently sized cannula, or in cases where it is advantageous to avoid placing a cannula in the ascending aorta in close proximity to the area of reconstruction. Also, it provides a means for antegrade cerebral perfusion in situations where extensive aortic reconstruction would otherwise necessitate prolonged periods of circulatory arrest. Our previous experience with 86 neonates operated on at the University of Oklahoma and University of Alabama at Birmingham demonstrated no postoperative distortion or thrombotic complications with the innominate artery [1], underscoring the safety of this approach.

Recently, we have expanded our utilization of SAC to include reoperative cases, where the risk of sternal reentry injuries is high, or in which inadvertent sternal reentry injuries have occurred requiring the initiation of CPB in infants not suitable for femoral cannulation. We have found it to be an extremely helpful adjunct to the congenital heart surgeon in reoperative complex cardiac surgery. The technique is simple, it does not require a separate incision, and the exposure is expeditious and can be life-saving in difficult re-entry situations. The technique may be used in older patients with equal ease, and may provide the congenital cardiac surgeon with a viable alternative strategy in the operating room.

## Competing interests

The author(s) declare that they have no competing interests.
